# Multi scale-aware attention for pyramid convolution network on finger vein recognition

**DOI:** 10.1038/s41598-023-50993-6

**Published:** 2024-01-04

**Authors:** Huijie Zhang, Weizhen Sun, Ling Lv

**Affiliations:** School of Biological Science & Medical Engineering, Southeast University, Nanjing, 210096 China

**Keywords:** Computational biology and bioinformatics, Image processing, Machine learning

## Abstract

In recent years, biometrics has been the most popular style of personal identification. The finger vein is an intrinsic and stable trait, and with the ability to detect liveness, it receives academic and industry attention. However, convolution neural networks (CNNs) based finger vein recognition generally can only cover a small input region by using small kernels. Hence, the performance is poor, facing low-quality finger vein images. It is a challenge to effectively use the critical feature of multi-scale for finger veins. In this article, we extract multi-scale features via pyramid convolution. We propose scale attention, namely, the scale-aware attention (SA) module, which enables dynamic adjustment of the weight of each scale to information aggregation. Utilize the complementation of different scale detail features to enhance the discriminativeness of extracted features, thus improving the finger vein recognition performance. In order to verify the present method’s efficiency, we carried out experiments on two public data sets and one internal data, and the wide range of experimental results proves the proposed method’s effectiveness.

## Introduction

Biometric identification is already widespread in everyday life, such as mobile payment, countries’ banking systems, entrance guard systems, etc. Finger veins traits are a unique biometric trait via irradiating near-infrared (NIR) light, which is located beneath the skin tissue layer, and the deoxygenated hemoglobin in the blood of the finger veins absorbs near-infrared light energy. The finger vein non-contact acquisition can also bring a preponderance of finger vein traits: in vivo detection and high security. As the superiorities of finger vein traits, it is a promising approach by researchers. However, the advancement of finger vein recognition has fallen far short of expectations. The performance of finger vein recognition is susceptible to extrinsic and intrinsic factors, such as user behavior, ambient lighting, and light scattering within the finger tissues. As a result, for finger vein recognition and verification, it is urgent to design high-precision and robust finger vein feature extraction algorithms.

More generally accepted, feature extraction is the crucial step for automatic person recognition using finger vein, as verification performance is sensitive to it. In order to improve the performance, there are methods of image pre-processing applied to moderate the noise and enhance image contrast, such as adaptive histogram equalization (CLAHE) before feature extraction. Feature extraction can be classified into feature extraction-based hand-crafted and feature extraction-based deep learning. Despite the experimental results of hand-crafted being satisfactory, it inevitably got into elaborate pre-processing steps to reinforce vein texture. Besides this, the generalization ability of such methods is limited, owing to customized designs for some particular databases, and thus, they are only sometimes applicable to emerging databases. In recent years, convolutional neural networks have made tremendous progress in feature capabilities and remarkable image quality robustness, widely used in computer visions. Recently, with the growth of deep learning and the need for privacy of personal authentication, biometrics identification-based deep learning has been the trend, especially finger vein recognition, which has been the focus of scholars. Hou et al.^1^ represent an auto-encoder based on Convolutional, which can learn high-level feature representation from raw finger vein images. Noh et al.^2^ proposed that finger vein recognition integrates shape images with texture images based on a densely connected convolutional network, which can efficiently use diverse features and reduce the sensitivity to noise. Du et al.^3^ proposed a method named FVSR-Net consisting of a multi-scale CNN and the improved biological optical model, which increases computational efficiency and stability. Fang et al.^4^ proposed finger vein recognition based on a dual-stream convolutional network combined with incorporating both the mini-ROI and the original image, which achieved superior results, Wang et al.^5^ presented a finger vein recognition approach with multi-receptive fields, which enhances the correlation of spaces and channels via dimensional interactive attention. Huang et al. designed an attention mechanism named joint attention (JA) to focus on the details of features, which dynamically adjusts the information aggregation in the spatial channels.

Inspired by these prior efforts and image detection, we presented an end-to-end backbone based on a novel mechanism for residual attention called “Scale-aware Attention (SA)”, which focuses on multi-receptive fields based on pyramid convolutional. The structure mainly consists of two stages: the feature extraction and the SA selection stages. In particular, it exploited pyramid convolutional to obtain different scale features from the input. This single-scale feature contains different details. Then, the SA choice stage leverages dynamic adjustment of the weights of different scale features to complement each feature. Finally, the refined feature was used for finger vein verification. The major contributions of the current work are outlined below:

1. We proposed a novel end-to-end model that replaces the stand convolutional with the pyramid convolutional. The proposed network uses a convolutional pyramid to extract multi-scale features via different kernel sizes to broaden receptive fields.
2. On the basis of SA, a coarse-to-fine feature extraction architecture is suggested which allows for end-to-end training, and the weights of different scale features can adjust adaptively. Using generalized mean pooling to generate the fused component weighted at different scales.
3. We conduct extensive experiments on public and in-house finger vein datasets, demonstrating proposed method outperforms existing SOTA methods while preserving good model and efficiency.

## Related work

### A brief review of pyramid convolution

Pyramid convolution is widely used in image classification since it uses different convolution kernels to expand the receptive field and complementary information of different scales. Wang et al.^6^ present a multi-scale pyramid Convolution module with spatial attention and channel attention to pay more attention to the images’ object. Liang et al.^7^ designed a model named SC2Net, which obtains the predicted density maps using residual pyramid dilated convolution (ResPyDConv). Jie et al.^8^ deliver a well-traced spatial pyramid module that gathers global and local cues to solve the challenge of scale variation in deep convolutional networks. Jia et al.^9^ combined pyramid-dilated convolutional blocks (PDCBs) with gated fusion units to reconstruct tiny image details since the PDCBs expand receptive fields and obtain details of the images via the network. Sun et al.^10^ proposed a pyramid attention mechanism for classifying the Hyperspectral Images (HIS) based octave convolution network. Bao et al.^11^ propose a residual attention pyramidal convolution for expression recognition-based VGG network, which uses CBAM attention to improve the weight of crucial aspects in multi-scale features extracted by pyramidal convolution. Moreover, it has better recognition performance and lightweight characteristics compared with other state-of-the-art networks. Due to the unique characteristics of finger vein images, like only grey channels and low contrast, extended receptive fields are a practical measure, and the studies on multi-receptive fields of finger veins mentioned in the previous section are successful example.

### Attention mechanism

The attention mechanism diverts more attention to essential regions and disregards irrelevant parts, imitating the human visual system. Moreover, it has exhibited excellent performance in various computer vision tasks, for instance, object detection, image classification, meta-learning, and individual recognition. Attention mechanisms can be categorized as temporal attention, spatial attention, channel attention, compound attention, and branch attention. SENet^12^ is the pioneered attention channel, which, via a squeeze-and-excitation (SE) block, includes a squeeze module and excitation module to improve representation ability by adjusting the weight of the channel-wise relationship. Spatial transformer networks (STN)^13^ are classical spatial attention systems that use a learnable module named spatial transformer to exploit spatial manipulation. Temporal attention is widely used for video processing to dynamically select when to pay attention. Selective kernel network (SKNet)^14^ is classical branch attention, which uses soft attention to be guided by the information in these branches. The convolutional block attention module (CBAM)^15^ represents compound attention, which recalibrates the significance of various spatial channels and positions by rescaling.

In finger vein authentication, there is much research about attention mechanisms. The attentions usually are a transformer of spatial attention, channel attention, and CBAM. As the multi-receptive field is extracted, the attention mechanism-based SKNet should be adequate for the multi-branch network, but it takes lots of computational power not applied. A new attention mechanism is designed from the scale level in this paper.

## Methodology

**Figure 1:**
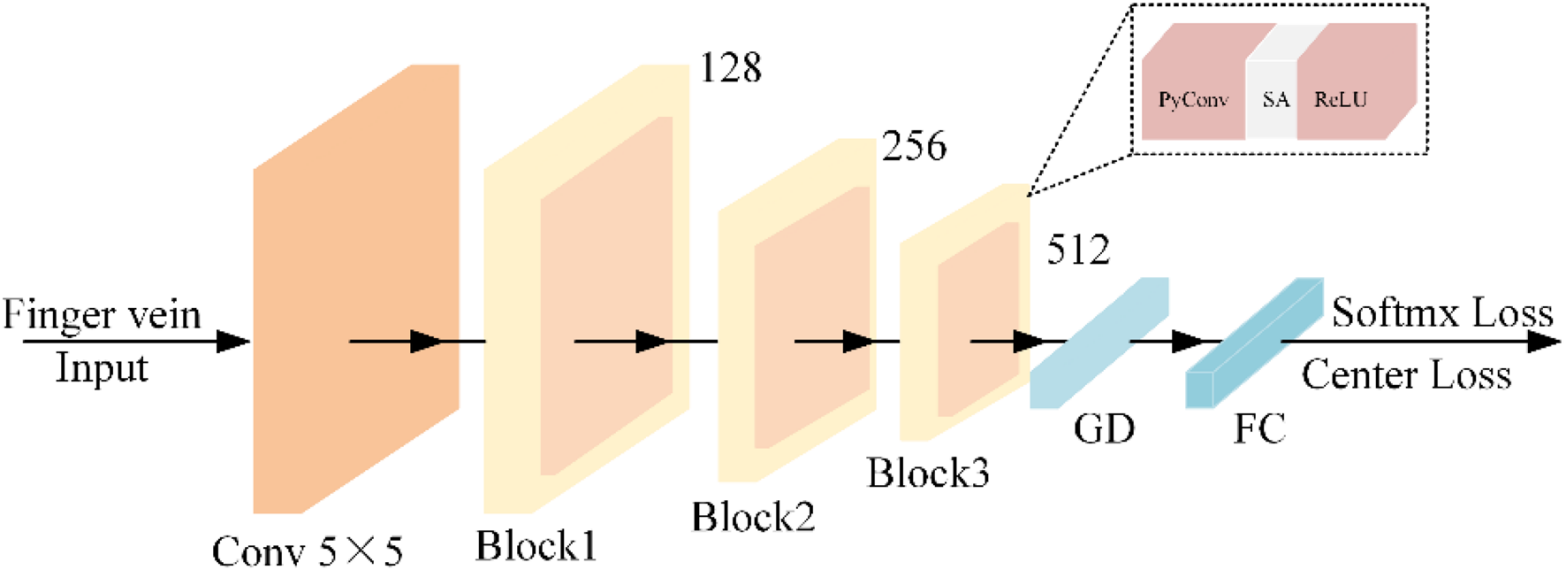
Overview of RSAFVNet.

**Figure 2:**
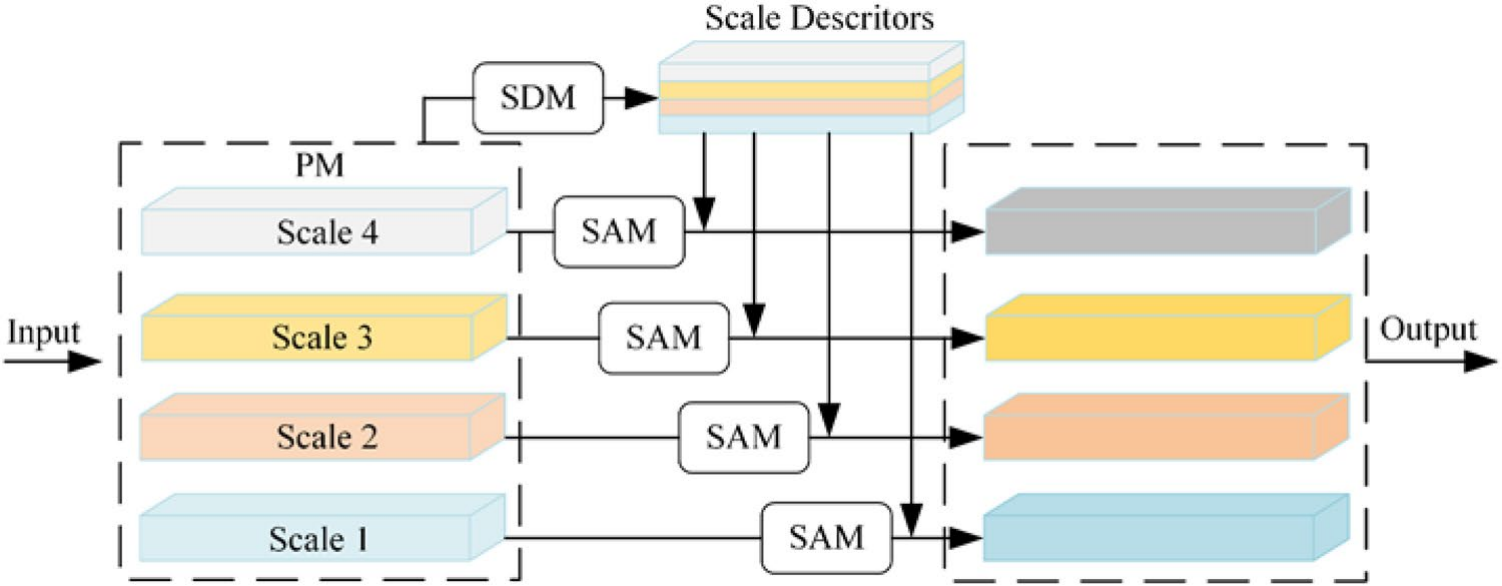
RASAPyConv’s structure.

This section presents a new finger vein identification network with Residual scale-aware attention, namely RSAFVNet. The main task of RSAFVNet is to capture the details with a multi-scale, which captures the intense discrimination with an end-to-end structure. Figure 1 gives an overview of the structure of RSAFVNet. We designed a backboned named Residual scale-aware attention Pyramidal Convolution (RSAPyConv), which uses pyramidal convolution instead of strand convolution to extract multi-scale finger vein feature. The specific RASPyConv module block structure, as shown in Fig. 2, mainly includes the pyramidal convolution, scale-aware attention that consists of scale descriptor and scale attention block, the global descriptor, and the loss function.

### Pyramidal convolution

**Figure 3:**
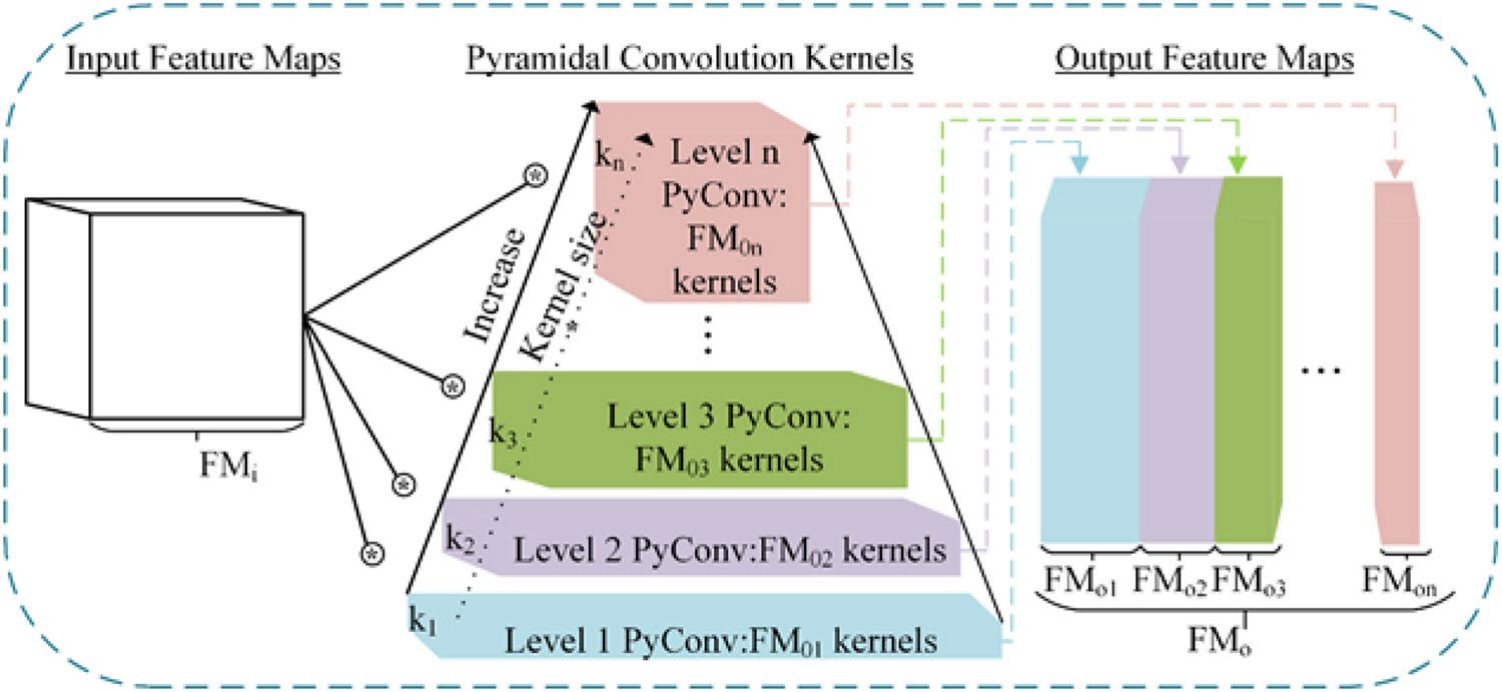
Pyramid convolution (PyConv) structure.

Convolutional neural networks are the workhorse for finger vein identification. The convolutional operation is the core of deep learning, which learns spatial kernels/filters for various computer vision tasks. Single kernel size standing convolution is used in most deep learning frameworks, which limits the network’s receptive field. To expand the receiving field of the kernel, multi-branch CNNs were proposed, such as the representative InceptionNet families, but the amount of calculation was increased. Duta^16^ proposed a multi-scale convolutional module named PyConv. An illustration is presented in Fig. 3, which displays a pyramid with n layers of various kernels, and the kernel has different spatial sizes for each level PyConv. With increasing the level of PyConv in the PyConv pyramid, the type of kernel size increased. In conjunction with the rise in space size, the depth of the kernel drops from level 1 to level n.

**Figure 4:**
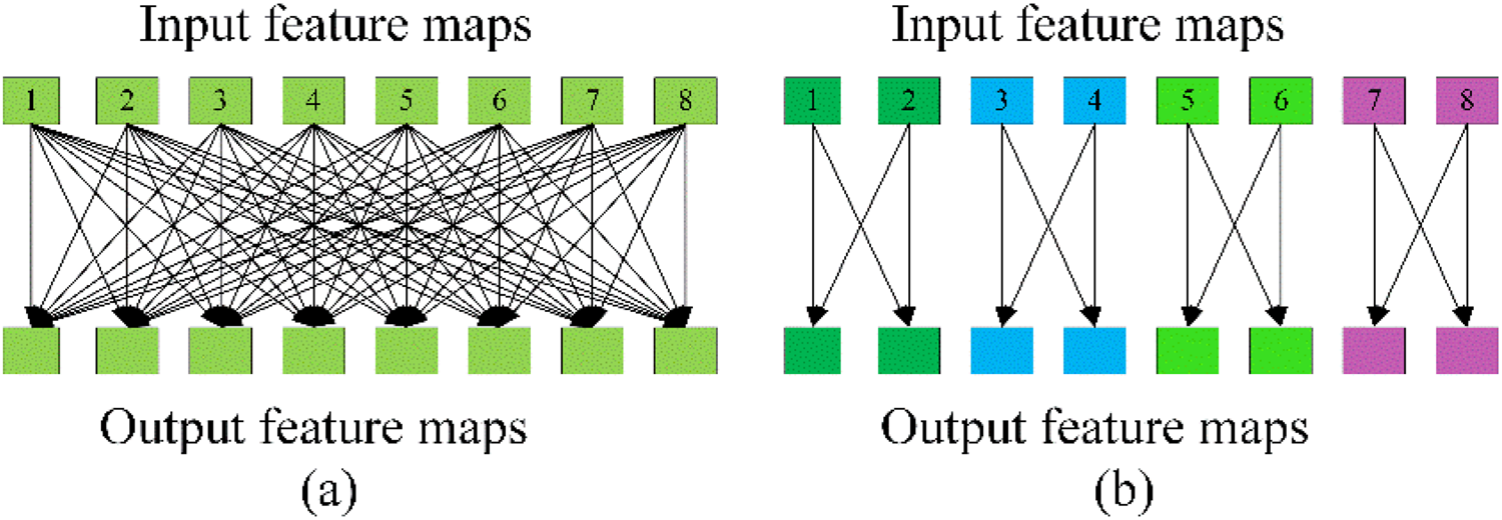
Group convolution.

A varying kernel depth is applied at each level, and the input feature maps FM_i_ were separated into different groups. Two examples of various convolutions are displayed in Fig. 4. This example has eight output and input feature maps. The standard convolution is illustrated in Fig. 4a, where the depth of the kernel was equal to the number of input graphs. Consequently, each output feature map was associated with all input feature maps. Figure 4b indicates that the input feature maps were classified into four groups, each with the same kernel size. As a result, the kernel depth was decreased by four. Moreover, grouped convolution could significantly decrease the computational cost and the number of convolution parameters. On this basis, we introduced the PyConv into finger vein verification. Additionally, the critical issue for feature maps at various scales is how to capture the feature response between scales at each level.

### Scale-aware attention

To our knowledge, the finger vein images are remarkably similar, so the feature extracted for finger vein recognition requires high visual expression ability to address the slight difference in types of finger veins; multi-scale receptive fields were extracted in pyramidal convolution. Directly fusing the different scale maps to learn more discriminative features will drown out the valuable information in the redundant information volume. In light of the above analysis, we presented the SA to adaptively exploit complementary characteristics at various scales produced by the pyramidal convolution. The suggested SA comprises a scale attention module (SAM) and a scale descriptor module (SDM). SDM uses the different group maps to generate the scale descriptor. Meanwhile, the SAM combines the level descriptor to refine the scale features.

#### Scale descriptor module

**Figure 5:**
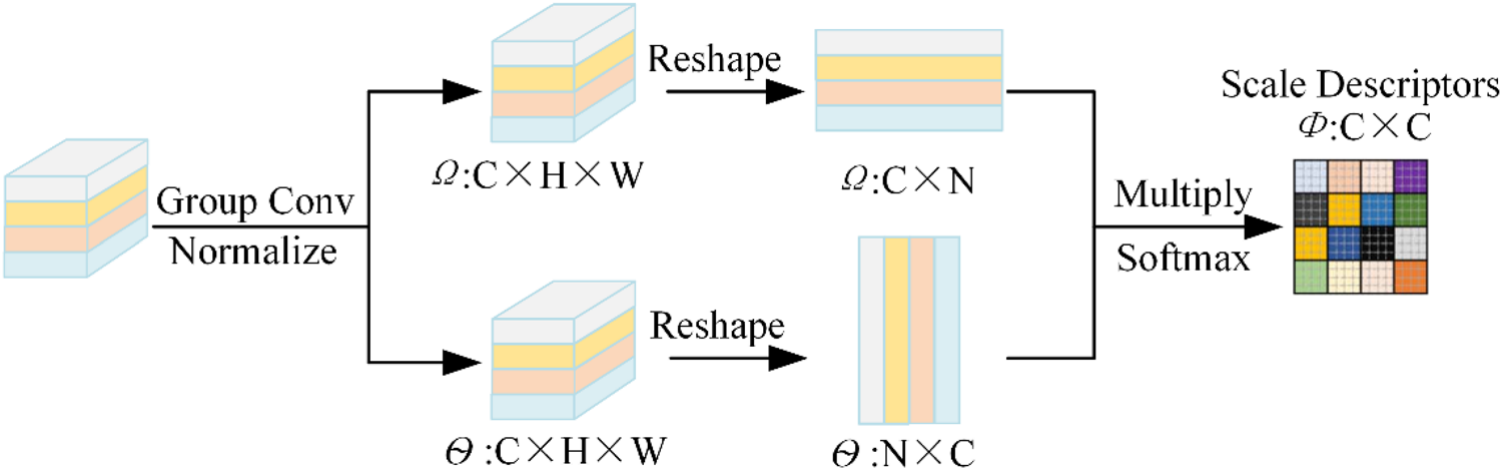
Detail of scale descriptor module.

The operational detail of SDM as illustration Fig. 5. The out of Pyramidal convolution includes multi-scale feature maps are fed into the SDM and the output is denoted as $\Omega \in {\mathbb{R}}^{4C \times H \times W}$ and $\Theta\, { = }\,{\mathbb{R}}^{4C \times H \times W}$. Firstly, SDM employs a $1 \times 1$ convolutional layer to decrease the channel sizes. Then the SDM reshapes the $\Omega$ to ${\mathbb{R}}^{C \times N}$, and $\Theta \in {\mathbb{R}}^{N \times C}$ where $N = H \times W$ represents the input finger vein image’s pixels number. And as the Pyramidal convolution uses four type of keral sizes by groups, $\Omega$ can be represented as $\Omega = [\Omega_{1} ,\Omega_{2} ,\Omega_{3} ,\Omega_{4} ]$, $\Theta$ can be represented as $\Theta { = }\left[ {\Theta_{1} ,\Theta_{2} ,\Theta_{3} ,\Theta_{4} } \right]$. The $\Omega_{i}$ and $\Theta_{i} \in {\mathbb{R}}^{{\frac{C}{4} \times N}}$ signal as the i-th scale. After that, SDM utilizes matrix multiplication between the transpose $\Theta$ and $\Omega$, which is subsequently normalized with softmax lay to produce the scale descriptors:

1 $$\begin{aligned} \Phi = & soft\max (\Omega \times \Theta ) \\ = & \left[ \begin{gathered} \Phi_{1} \hfill \\ \vdots \hfill \\ \Phi_{4} \hfill \\ \end{gathered} \right] = \left[ \begin{gathered} \Phi_{11} \cdots \Phi_{14} \hfill \\ \vdots \ddots \vdots \hfill \\ \Phi_{41} \cdots \Phi_{44} \hfill \\ \end{gathered} \right], \\ \end{aligned}$$

where $\Phi_{ij} = \Omega_{i} \Theta_{j}$. As a result, the $\Phi$ contains the information on the details of all scales. In this way, the scale descriptors produced by SDM contain both location and multi-scale information.

#### Scale attention module

**Figure 6:**
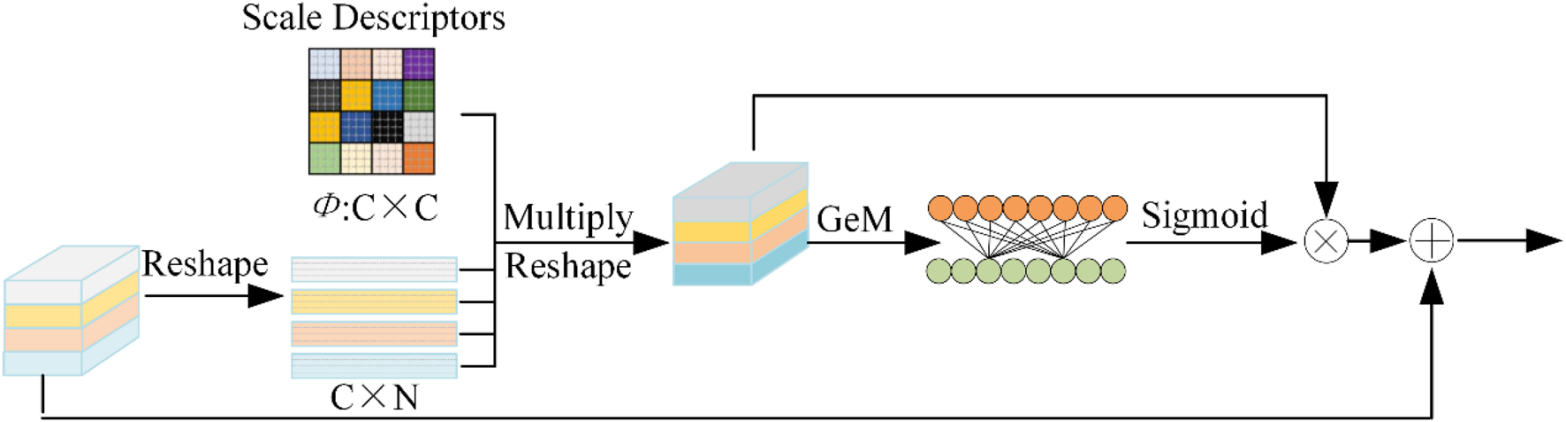
Details of scale attention module.

We argue that single-scale features lack adequate semantic or spatial detail information to gain the performance of finger vein authentication. Hence, multi-scale information in the scale descriptors can augment single-scale features to give them greater discriminative power. In particular, as illustrated in Fig. 6, given the level descriptors $\Phi$ and $F_{i}$, $i \in \left[ {1,2,3,4} \right]$, SAM adopts a matrix multiplication method to perform the perception of multi-scale information. Consequently, SAM acquires attentional scale characteristics with scale awareness. To further strengthen the feature representation, SAM applies a skip-connection approach here.

### Analysis performance of RASAPyConv

RASAPyConv is the core of the design. According to the structure of Fig. 2, it can be known that during the feature extraction process, the input is first through the pyramidal convolution module, which generates multi-scale feature maps. Then, it is compressed by point convolution to bring the number of channels down to the number of single-scale channels, reducing network parameters. Finally, the feature maps through the SA attention mechanism are combined for dimension expansion. SA is mostly matrix transformation, so the central computation of the RASAPyConv module is generated by the PyConv module. The PyConv module is analyzed from two aspects: the number of parameters (space performance) and the computational requirements Floating Point Operations) (FLOPs) (time performance).

Assume that the PyConv module has *FM*_*i*_ as the number of input feature channels, the dimensions of each convolution layer are {$K_{1}^{2}$, $K_{2}^{2}$, $K_{3}^{2}$, $K_{4}^{2}$}, and the convolution depth is $\left\{ {FM_{i} ,FM_{i} \left( {\frac{{K_{1}^{2} }}{{K_{2}^{2} }}} \right),FM_{i} \left( {\frac{{K_{1}^{2} }}{{K_{3}^{2} }}} \right),FM_{i} \left( {\frac{{K_{1}^{2} }}{{K_{4}^{2} }}} \right)} \right\}$ respectively, the number of feature channels output by convolution in each layer is ${{\{ FM}}_{o1} {,}{\text{FM}}_{o2} {,}{\text{FM}}_{o3} ,{\text{FM}}_{o4} {{\} }}$ respectively, and the number of parameters together with FLOPs corresponding to PyConv module is as below:

2 $$\begin{aligned} parameters = & K_{1}^{2} FM_{i} FM_{o1} + K_{2}^{2} FM_{i} \left( {\frac{{K_{1}^{2} }}{{K_{2}^{2} }}} \right)FM_{o2} \\ & + K_{3}^{2} FM_{i} \left( {\frac{{K_{1}^{2} }}{{K_{3}^{2} }}} \right)FM_{o3} + K_{4}^{2} FM_{i} \left( {\frac{{K_{1}^{2} }}{{K_{4}^{2} }}} \right)FM_{o4} , \\ \end{aligned}$$

3 $$\begin{aligned} FLOPs = & K_{1}^{2} FM_{i} FM_{o1} (H \times W) + K_{2}^{2} FM_{i} \left( {\frac{{K_{1}^{2} }}{{K_{2}^{2} }}} \right)FM_{o2} (H \times W) \\ & + K_{3}^{2} FM_{i} \left( {\frac{{K_{1}^{2} }}{{K_{3}^{2} }}} \right)FM_{o3} (H \times W) + K_{4}^{2} FM_{i} \left( {\frac{{K_{1}^{2} }}{{K_{4}^{2} }}} \right)FM_{o4} (H \times W). \\ \end{aligned}$$

Among them, each of the four additive terms on the right-hand side of the equation corresponds to the number of parameters and the amount of computation for each layer of convolution in the PyConv module, $\left( {\frac{{K_{1}^{2} }}{{K_{i}^{2} }}} \right)$ a represents the number of groups of grouped convolutions of this convolutional layer, $i = 1,2,3,4$.

Through formulation simplification, it can be found that although the size of the convolution kernel in the PyConv module increases from $K_{1}^{2}$ to $K_{4}^{2}$, the parameters and calculation amount of each layer of convolution are equal to the parameters and calculation amount of the standard convolution of size $K_{1}^{2}$, which makes the PyConv module to expand the receptive domain of convolution kernel and improve the network performance without increasing the computational overhead. Therefore, compared with standard convolution, PM module has the following advantages.

1. *Multi-scale processing* The PyConv module has convolution kernels of different sizes and depths, which can parse input features from multiple scales and promote the full expression of finger vein features by fusing multi-scale features.
2. *High efficiency* The convolution of each layer of the PyConv module can realize independent parallel calculation, even run independently on different machines, and perform feature fusion. Hence, the overall calculation efficiency is high.

### Global feature descriptor

For biometrics, the authentication is to obtain a score that a feature vector extracted from the biometric traits’ enrollment data compared with the obtaining. This is consistent with the image retrieval domain, which uses the global feature descriptor. Compared with the local features, it summarizes an image’s content as a compact representation, and similarity learning is not limited by the object posed and the image’s qualities. As those limitations also influence the performance of finger vein identification and the high similarity of local texture features, we employed global feature descriptors to produce a compact feature with multi-scale receptive fields for finger vein authentication.

GeM pooling can increase the features’ representability via a nonlinear learnable operator, so it has been adopted for biometrics identification tasks^17^. Here, for a specific input finger vein image, the output of the RSAPyConv layer is a 3-dimensional tensor X with $C \times W \times H$ size, indicating the number of channels, weight and height of feature maps separately. In computer vision, global max pooling (MAC^18,19^) and global average pooling (SPoC^20^) are generalized GeM pooling, which can be described as the below formulation:

4 $$f^{(g)} = [f_{1}^{(g)} \cdots f_{c}^{(g)} \cdots f_{C}^{(g)} ],f_{c}^{(g)} = \left( {\frac{1}{{\left| {X_{C} } \right|}}\sum\limits_{{x \in X_{C} }} {x^{{P_{C} }} } } \right)^{{\frac{1}{{P_{C} }}}} .$$

In which, the pooling parameter $P_{c}$ is a learnable parameter, when $p_{c} \to \infty$ or $p_{c} = 1$, the given form is the same as MAC or SPoC separately. The configurable and shareable pooling layer parameters allow for a highly non-linear presentation to be achieved with varying pooling parameters $p_{c}$. What is more, the GeM pooling operation is divisible and can be optimized via back-propagation, guaranteeing that our presented RSAFVNet can be trained end-to-end.

### Joint loss function strategy

The feature representation with small intra-class and significant inter-class distances can better promote finger vein recognition. In our experiment, a joint loss function, which combines softmax classification loss with a deep metric-based loss function (Center Loss function), was applied. Since the softmax loss only forces expand the inter-class distance of different classes, ignoring the intraclass distance, the finger veins’ position and the lights may lead to a significant difference of the same finger vein in the capture, so that it may cause misclassification. The center loss function has a better ability to reduce the intraclass distance strongly, so we introduce it into the network, and the calculation process is as follows:

5 $$L_{{C}} = \frac{1}{2}\sum\limits_{i = 1}^{m} {\left\| {x_{i} - c_{{y_{i} }} } \right\|} ,$$

where $x$ represents the input features, $c_{i}$ denotes the center of all samples that have the same class label as $y_{i}$.

Therefore, the joint loss function calculation process is as follows:

6 $$L = L_{s} + \lambda L{}_{c},$$

where λ is used to keep them in balance. And we will discuss its influence in the following experimental section.

## Experimental study

This section briefly describes the finger vein datasets utilized, the two public finger vein datasets, and an internal dataset. This section also describes the metrics that were evaluated. Then, it illustrates the detailed implementation, including default settings and training data preparation. Lastly, many experiments and their contents are employed to prove the effectiveness of the method presented, which includes ablation experiments, compared to other attention methods. At the same time, the superior performance of the presented method was proved compared to other state-of-the-art methods on a public benchmark dataset.

### Datasets and evaluation protocol

#### Datasets

- *FV-USM (USM)* The FV-USM^21^ dataset includes 2952 images at all, which was conducted via Universiti Sains Malaysia, George Town, Malaysia. Finger vein images were collected from the middle and index fingers of 123 people’s hands, and they were gathered six times for each finger. The original images are grayscale and $640 \times 480$ resolution. Also, there is a resolution of 100 × 300 for the ROI provided in this dataset.
- *SDUMLA-HMT (SDUMLA)* The SDUMLA^22^ datasets includes 3816 images in all, which was conducted via Shandong University, Jinan, China. Finger vein images were collected from the ring, middle and index fingers of 106 people on both hands, and they were taken six times for each finger. The original images are grayscale and 320 × 240 resolution.
- *GERWIN* The GERWIN^23^ datasets includes 8316 images in all, which was performed by our team. Finger vein images were collected from the ring, middle and index fingers of 106 people on both hands, and they were taken six times for each finger. The original images are grayscale and 600 × 200 resolution.

#### Evaluation protocol

In evaluation protocol, we adopt genuine pairs and impostor pairs, which introduced in^24^. Expressed mathematically as:

7 $$N_{g} = C_{{N_{f} }}^{2} N_{c} = N_{c} N_{f} (N_{f} - 1)/2,$$

8 $$N_{i} = {\text{C}}_{{N_{C} }}^{2} ,$$

where the Ng represents the number of genuine pairs, the Ni represents the number of imposto pairs. Nc represents the number of finger vein categories in test set and Nf indicates the number of samples per finger vein category.

In order to fairly assess the behavior of various methods, equal error rate (EER) and accuracy (Acc) were calculated on a subset of tests. EER value equals the value when the false acceptance rate (FAR) equals the false rejection rate (FRR). The algorithms with a lower EER demonstrated improved performance on the validation tasks.

9 $$FAR = \frac{Number\,of\,genuinere\,rejection}{{Number\,of\,genuine\,attempted}},$$

10 $$FRR = \frac{Number\,of\,imposter\,rejection}{{Number\,of\,genuine\,attempted}}.$$

The open-set protocol was used in our experiment to assess the behavior of finger-vein verification, which is irrelevant to the actual application since the training classes and test classes do not overlap. In particular, for each database, one-half of the classes are randomized for training, and the remainder are applied for testing. In our trials, a fourfold cross-validation method was employed.

### Implementation details

The above datasets have different resolutions, with redundant information and background fixed noise. We extracted the ROI image from those original images as the method applied in^25^ and resized the finger vein samples to 224 × 224 × 3 for training. The grayscale one-channel sample was expanded to a three-channel image by copying twice as input. In^26^ tradition, national data augmentation can improve the performance of finger veins and prevent overfitting. In our experiment, we adopt data augmentation methods such as flip and shear, etc., the specific ones listed in Table 1, which gives greater flexibility to train samples within each training cycle and to train finger vein samples without increase. The training samples of input are transformed randomly prior to entering each training epoch. In this format, the number of training samples remains constant.

**Table 1:** Data augmentation parameters.

Methods	Parameters	
Flip	Vertical	True
	Horizontal	False
Rotation (degree range for random rotation		2.5
Shift (range scale in [− 1, 1])	Width	0.05
	Height	0.05
Shear range (range in [− 1,1])		0.05
Zoom (range in [1 − zoom, 1 + zoom range])		0.05

In our experiments, the parameters are designed on the basis of previous experience of finger vein identification based on deep learning. The input batch size is 128, which includes 32 subjects and four samples in each subject. Furthermore, the epoch maximum is 100. The optimizer is an Adaptive Moment Estimation with an initial learning rate of 0.001 and dynamically adjusts using the ReduceLROnPlateau function. Specifically, the learning rate decreased to the original 0.1 when the validation set loss was not reduced within 20 batches. It should be pointed out that the experiment was conducted utilizing Python with the PyTorch frame on a workspace computer that had the below specifications: Intel(R)Core(TM) i7-8700 CPU @ 3.20 GHz RAM 16 GB, and GPU NVIDIA GeForce GTX 1060 6 GB.

### Analysis of joint loss function strategy

#### Parameter selection

In order to assess the parameter λ performance of the network presented, in our tests, the scope of λ is between 0.01 and 5. The mean value was applied to determine the performance of the presented method, see Table 2.

**Table 2:** Performance of different loss functions in different datasets.

λ	FV-USM		SDUMLA-HMT		GERWIN	
	Acc (%)	EER (%)	Acc (%)	EER (%)	Acc (%)	EER (%)
0.01	99.86	1.35	96.38	4.92	97.56	3.65
0.05	99.89	1.22	97.02	2.31	98.87	2.86
0.1	99.92	1.02	98.70	1.30	99.10	1.32
0.5	99.91	1.06	98.95	0.73	99.05	0.65
1	99.91	1.10	98.15	0.95	99.08	0.36
2	99.90	1.12	97.92	1.45	99.16	0.23
5	99.90	1.12	97.95	1.64	98.52	2.32

Table 2 displays the performance of the presented method in various datasets. This indicates that the suitability of the parameter λ can improve the recognition of finger veins. For instance, in the FVUSM dataset, the optimum performance of the presented method is achieved with λ equals to 0.1, the Acc of 99.92%, and EER of 1.02%. Similarly, in the SDU datasets, the optimum performance of the presented method with the λ equal 0.5, the Acc of 98.95%, and the EER of 0.73%. As for the GERWIN dataset, the proposed method’s best performance with the λ equals 2, the Acc of 99.16%, and EER of 0.23%.

#### Loss function comparison

To assess the effectiveness of the joint loss function strategy, softmax, and the central loss function were compared to the joint loss function. The performance of different loss functions is listed in Table 3 in detail.

**Table 3:** Performance comparison of different loss functions.

Loss function	FV-USM		SDUMLA-HMT		GERWIN	
	Acc (%)	EER (%)	Acc (%)	EER (%)	Acc (%)	EER (%)
Softmax loss	98.18	2.35	96.83	4.29	98.16	3.65
Center loss^27^	99.40	1.42	98.02	1.31	98.87	1.68
The proposed loss	99.92	1.02	98.95	0.73	99.16	0.23

As illustrated in Table 3, the properties of the joint loss function-based FV-USM dataset are compared to the softmax loss and central loss functions; the Acc increased by 1.74% and 0.52%, and the EER increased by 1.33% and 0.40%, respectively. The performance of the SDUMLA-HMT datasets based on the joint loss function compared to the softmax loss and central loss functions, the Acc increased by 3.12% and 0.93%, and the ERR increased by 3.56% and 0.58%, respectively. The performance of the GERWIN datasets based on the joint loss function compared to the softmax loss and central loss functions, the Acc increased by 1.00% and 0.29%, and the ERR increased by 3.52% and 1.45%, respectively.

### Effectiveness of the pyramidal convolution

An experiment was performed on SDUMLA-HMT, FV-USM, and GERWIN datasets to validate the significance of the pyramidal convolution. The pyramidal convolution was replaced by standard convolution, which has the same number of channels. The output of the standard convolution reduces the channel to one in four and splits it into four groups, achieving the scale-aware attention mechanism with a single scale. The performance of different convolutions is listed in Table 4. As shown, the pyramidal convolution outperforms the standard convolution.

**Table 4:** Performance comparison of different convolutions.

Convolution	FV-USM		SDUMLA-HMT		GERWIN	
	Acc (%)	EER (%)	Acc (%)	EER (%)	Acc (%)	EER (%)
Standard	99.35	1.32	87.76	3.73	89.34	6.56
Pyramidal^16^	99.92	1.02	98.95	0.73	99.16	0.23

### Effectiveness of the SA attention module

#### Ablation study of SA attention

In order to investigate the properties of the proposed module of SA, an ablation study of SA was performed on datasets of SDUMLA-HMT, FV-USM, and GERWIN. Furthermore, SA attention evolved from PA attention, contrasting with PA attention. The findings of the SA ablation study are presented in Table 5.

**Table 5:** Performance of SA.

Setting	FV-USM		SDUMLA-HMT		GERWIN	
	Acc (%)	EER (%)	Acc (%)	EER (%)	Acc (%)	EER (%)
No attention	99.03	1.54	97.67	1.37	97.54	2.36
+ PA	99.46	1.21	98.87	1.12	98.69	0.40
+ SA(ours)	99.92	1.02	98.95	0.73	99.16	0.23

As shown in Table 5, the performance of the FV-USM datasets with SA attention compared with no attention and PA attention increased by 0.89% and 0.46%, and EER increased by 0.52% and 0.19%, respectively. The performance of the SDUMLA-HMT datasets with SA attention compared with no attention and PA attention, the Acc increased by 1.82% and 0.08%, and ERR increased by 0.64% and 0.39%, respectively. Compared with no attention and PA attention, the performance of the GERWIN datasets with SA attention increased by 1.62% and 0.47%, and ERR increased by 1.13% and 0.17%, respectively.

#### Compared with different attention

**Figure 7:**
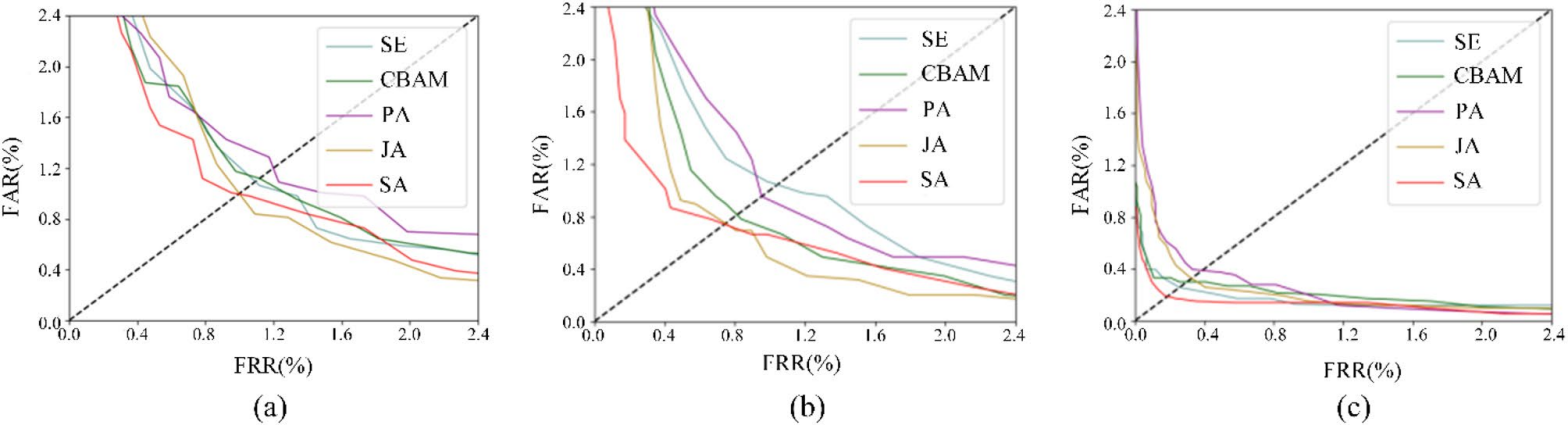
Comparison of EER for attention modules in various databases. (**a**) SDUMLA-HMT. (**b**) FV-USM. (**c**) GERWIN.

To better demonstrate the effectiveness of the presented scheme, two classical attentional mechanisms, CBAM and SE, were compared with the newly presented finger-vein joint attention. The optimum values are represented in bold, as illustrated in Table 6. From these results, each attention module outperforms the baseline modules with no attention. Nevertheless, the module with SA attention presents the best performance on finger vein public datasets and in-house datasets with the highest Acc and the lowest EER. These present the validity of the method proposed. Figure 7 displays the findings of the two former experiments, where performance is better as the curve gets closer to the axis, and the intersection point between the diagonal and the curve is the EER.

**Table 6:** Comparisons of different attention modules.

Setting	FV-USM		SDUMLA-HMT		GERWIN	
	Acc (%)	EER (%)	Acc (%)	EER (%)	Acc (%)	EER (%)
No attention	99.03	1.54	97.67	1.37	97.54	2.36
+ SE	98.14	1.12	98.12	1.15	98.35	0.28
+ CBAM	98.56	1.15	95.65	0.81	99.03	0.30
+ JA^17^	99.67	1.04	98.92	0.75	99.12	0.31
+ SA(ours)	**99.92**	**1.02**	**98.95**	**0.73**	**99.16**	**0.23**

### Comparison with SOTA methods

We assessed our presented network through a comparison with state-of-the-art finger vein authentication approaches. The tests were performed on the public datasets SDUMLA and FV-USM. The findings are displayed in Tables 7 and 8, and the bold indicates the optimum value.

**Table 7:** Comparisons with the state-of-art on SDUMLA-HMT.

Paper	Method	SDUMLA-HMT	
		Acc (%)	EER (%)
Li et al.^28^	Compact multirepresentation feature descriptor	96.43 $\pm$ + 2.45	–
Fang et al.^29^	Double-weighted group Spare representation	70.56	–
Dev et al.^30^	Multi Biometric finger vein template	99.03	3.87
Liu et al.^31^	Finger vein recognition based MMRAN	98.11	0.44
Huang et al.^32^	FVT	97.90	1.50
Vida et al.^33^	Cuboc-LBP	98.50	1.35
Ours	Scale-aware attention network	**98.95**	**0.73**

**Table 8:** Comparisons with the state-of-art on FV-USM.

Paper	Method	FV-USM	
		Acc (%)	EER (%)
Li et al.^28^	Compact multirepresentation feature descriptor	97.69 $\pm$ + 2.95	–
Fang et al.^29^	Double-weighted group Spare representation	97.00	–
Liu et al.^31^	Finger vein recognition based MMRAN	99.75	0.50
Vida et al.^33^	Cuboc-LBP	99.50	0.95
Huang et al.^32^	FVT	99.73	0.44
Ours	Scale-aware attention network	**99.92**	**1.02**

As we can see from the tables, our method has a slight advantage over the method proposed in Ref.^28^, and the method of extracted feature proposed does not need prior knowledge. Fang et al. proposed a double-weighted group sparse representation classification with some finger vein details lost, while our proposed method to extract multi-scale finger vein details feature. Liu et al.^31^ proposed attention by connecting a residual block with multistage residual attention. They achieved a good performance in public datasets, and Huang et al.^32^ presented a new transformer based on finger vein recognition, focusing on global information. However, the proposed method is superior to MMRAN and FVT, and the design of the finger vein model of the end-to-end multi-scale attention mechanism in this paper is also inspired by them. Overall, the proposed method has the lowest EER and the highest Acc of the compared methods, which indicates that the proposed method is effective.

## Conclusion

This paper presented a novel end-to-end finger vein authentication network based on the pyramidal convolution with a scale-aware attention module block. Pyramid convolution learned finger vein features at different scales, and the scale-aware captured various detailed features with multi-scale, avoiding the redundancy of finger vein features. By jointly applying the central loss and softmax loss functions, the inter-class clustering was increased, and the distance of the intraclass was reduced. The model’s effectiveness was verified on the open-source finger vein dataset and our in-house dataset. The ablation experiment demonstrated the effectiveness of the pyramid convolution module, joint loss function stagey, and the scale-aware attention mechanism module on recognizing the finger vein.

Although the proposed method performs significantly in finger vein authentication, some issues still need further research. Firstly, the finger vein collected will be mixed with noise in the collection process to extract more compelling features and prevent illegal people from introducing potential backdoor attack threats. Second, different types of finger vein data are collected in different usage scenarios, which requires the model to have high generalization performance. Still, future work can consider cross-validation experiments to contribute to the optimization of model parameters and improve the generalization capability. Finally, with the increase of users, the query authentication time will also increase significantly, so more convenient authentication methods are also a focus of future research.

## Data Availability

The data that support the findings of this study are available from SDUMLA (https://time.sdu.edu.cn/kycg/gksjk.htm) and FV-USM (http://drfendi.com/fv_usm_database/) but restrictions apply to the availability of these data, which were used under license for the current study, and so are not publicly available. Data are however available from the corresponding author upon reasonable request and with permission of official. GERWIN dataset generated and analysed during the current study are not publicly available due the requirements of all the subjects but are available from the corresponding author on reasonable request.
